# The role of circadian rhythms in the pathogenesis of myopia

**DOI:** 10.3389/fphys.2026.1797489

**Published:** 2026-04-01

**Authors:** Ning Zhang, Guoge Han, Rui Hao

**Affiliations:** 1 Clinical College of Ophthalmology, Tianjin Medical University, Tianjin, China; 2 Tianjin Key Lab of Ophthalmology and Vision Science, Tianjin Eye Hospital, Tianjin, China; 3 Nankai University Eye Institute, Nankai University Affiliated Eye Hospital, Nankai University, Tianjin, China

**Keywords:** circadian rhythms, dopamine, ipRGCs, melanopsin, myopia

## Abstract

Myopia is a complex ocular disorder arising from the interaction of genetic predisposition and environmental cues that regulate eye growth. Increasing evidence indicates that the circadian timing system plays a critical role in ocular development and refractive homeostasis. The retina, choroid, and sclera possess intrinsic molecular clocks that generate rhythmic oscillations in gene expression, neurotransmitter release, and tissue physiology. Disruption of these ocular circadian rhythms has been implicated in abnormal axial elongation through pathways involving dopamine and melatonin signaling, light-dependent retinal pathways, and diurnal fluctuations in intraocular pressure. In this review, we summarize current knowledge of the molecular mechanisms underlying circadian regulation in ocular tissues and discuss how environmental light exposure and sleep–wake cycles modulate these processes. We further integrate evidence linking rhythmic alterations in retinal, choroidal, and scleral function to myopia development. Finally, we propose mechanistic frameworks through which circadian dysregulation may contribute to myopia onset and progression, highlighting potential molecular targets for rhythm-based intervention strategies.

## 1 Background

Myopia is a condition in which parallel light rays focus in front of the retina, resulting in blurred vision. This condition is generally attributed to the excessive elongation of the eyeball that occurs during childhood. Presently, approximately 2 billion people worldwide are affected by myopia (28.3% of the global population), with about 277 million suffering from high myopia (4.0% of the global population). Projections indicate that by the year 2050, the global population of myopic individuals will reach 4.76 billion, accounting for 49.8% of the total global population. Furthermore, projections indicate that the prevalence of high myopia will reach nearly 1 billion cases, accounting for 9.8% of the world’s total population (Holden et al., 2016). Myopia has been demonstrated to significantly increase the risk of pathological changes in ocular tissues, notably for high myopia cases characterized by a spherical equivalent (SE) is lower than -6.0 D or when accompanied by secondary retinal pathology (pathological myopia). This further elevates the risk of developing blinding eye diseases (such as glaucoma, retinal detachment, and macular holes) (Bullimore and Brennan, 2019; Du et al., 2021; Zhang et al., 2024).

Despite the fact that the etiology of myopia remains incompletely understood, the preponderance of evidence indicates that both genetic and environmental factors play significant roles in its onset and progression (Cai et al., 2019; Harb and Wildsoet, 2019). These factors encompass seasonal transitions, meteorological variations, and the periodic changes in light exposure regulated by circadian rhythms (Mandel et al., 2008; Wirz-Justice et al., 2021). In recent years, the circadian rhythm system has been increasingly incorporated into the domain of ophthalmology as a crucial mechanism for regulating physiological functions in living organisms. Research indicates that dopamine and melatonin in the retina exhibit significant circadian oscillations, with these neurotransmitters playing a crucial role in inhibiting abnormal axial elongation of the eye (Stone et al., 2013; Chakraborty et al., 2021). Concurrently, experimental studies on animals have demonstrated that disrupting the light-dark cycle or clock gene expression accelerates axial growth and induces myopia (Nickla and Totonelly, 2016). These findings suggest that circadian rhythms may contribute significantly to myopia development in ways that have yet to be fully explored.

Therefore, this paper will elaborate on the following three levels: 1. An overview of circadian rhythms in myopia; 2. Rhythmic characteristics of different ocular structures (such as the retina, choroid, and sclera) and their association with myopia; 3. The pathway integration of light signals—molecular mechanisms—ocular axis regulation. Through this three-step systematic review, we aim to provide a comprehensive circadian perspective for myopia pathogenesis research and lay the theoretical foundation for future circadian-oriented intervention strategies.

## 2 Overview of circadian rhythms

### 2.1 Molecular basis of circadian rhythms in the eye

#### 2.1.1 Circadian rhythms and the biological clock

Circadian rhythms refer to the physiological and behavioral processes that occur in living organisms in cycles of approximately 24 hours. Such rhythms are mainly controlled by the central biological clock, a key pacemaker situated in the hypothalamus’s suprachiasmatic nucleus (SCN) (Welsh et al., 2010; Patke et al., 2020).

**Figure 1:**
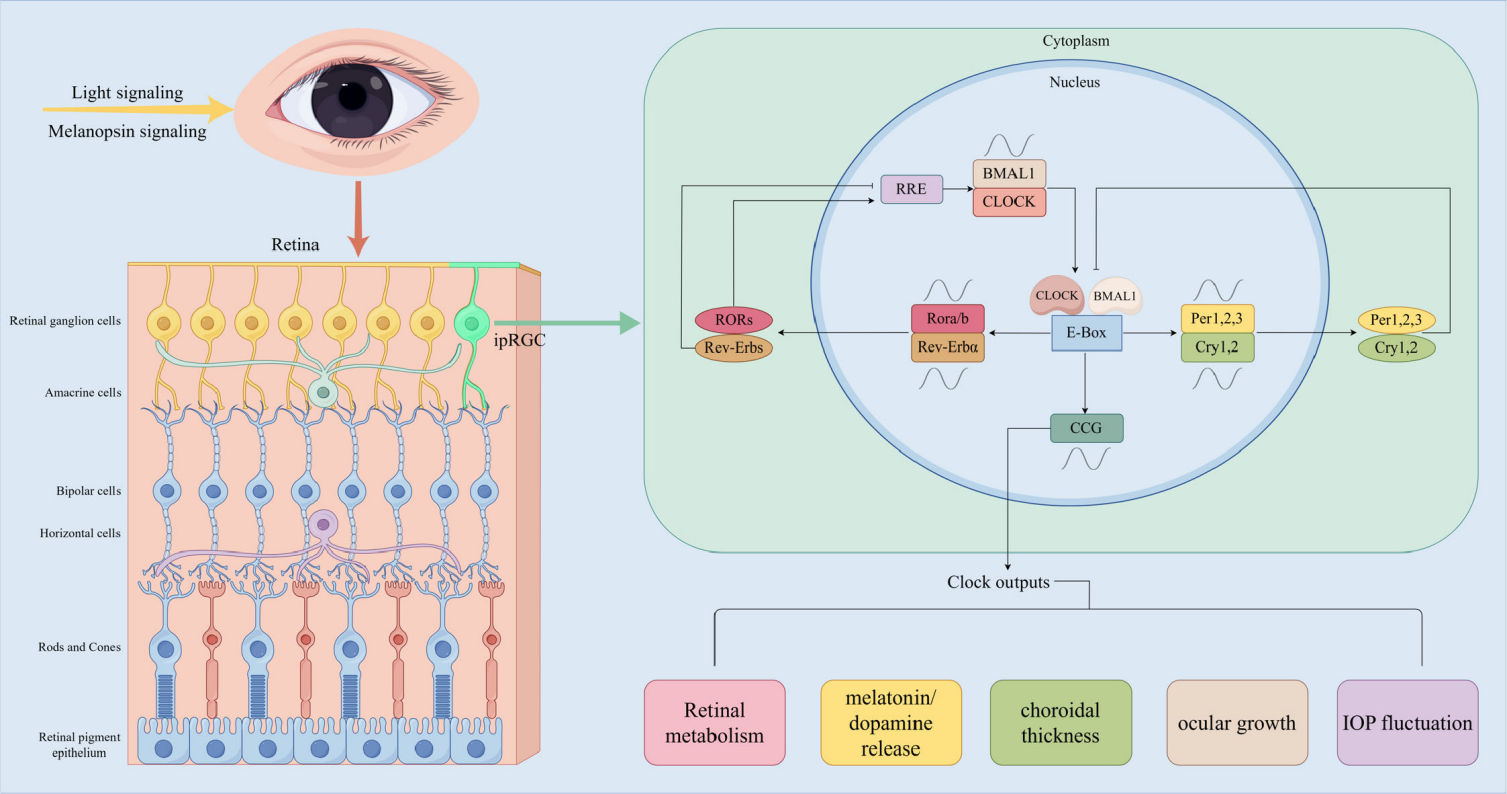
Schematic diagram of the molecular mechanisms of ocular circadian rhythms: ipRGCs mediate light signal input, and the core TTFL loop regulates clock gene expression, ultimately controlling retinal metabolism, neurotransmitter release, and ocular growth. BMAL1, Brain and muscle ARNT-like 1; CLOCK, Circadian locomotor output cycles kaput; Per, Period; Cry, Cryptochrome; ipRGCs, Intrinsically photosensitive retinal ganglion cells; TTFL, Transcription-translation feedback loops. By Figdraw.

The core molecular mechanisms of the mammalian circadian clock are contingent upon three interconnected transcription-translation feedback loops (TTFL) (**Figure 1**). Inside the core molecular circuit, the CLOCK/BMAL1 heterodimeric complex interacts with the E-box element, which in turn drives the cyclical transcription of Per1/2/3 (period) and Cry1/2 (cryptochrome) genes. Subsequently, the CRYPTOCHROME and PERIOD proteins enter the nucleus, where they inhibit CLOCK/BMAL1 activity, thus establishing a self-regulating oscillation with a duration of approximately 24 hours.

The second loop is responsible for the regulation of the periodic expression of Bmal1 by activating Rev-Erbα and Rora proteins. These proteins bind to the Ror/Rev-erb (RRE) elements in the Bmal1 promoter in a competitive manner, thereby stabilizing the amplitude of the core oscillator (Takahashi, 2015).

This molecular mechanism is conserved in the brain’s central biological clock and peripheral organs (e.g., liver, muscles), as well as in ocular tissues (retina, choroid, sclera, cornea) that harbor independent circadian regulatory systems (Reppert and Weaver, 2002). The retina, choroid, sclera, and cornea exhibit rhythmic expression of molecular clock genes (McMahon et al., 2014). **Figure 1** outlines the core molecular mechanism of the ocular circadian clock, which is based on two interconnected transcription-translation feedback loops (TTFL). The output of this clock system regulates key ocular physiological processes: retinal metabolism, rhythmic secretion of melatonin/dopamine, choroidal thickness fluctuations, and intraocular pressure homeostasis — all of which are directly or indirectly involved in refractive development. Disruption of any link in this loop (e.g., abnormal expression of BMAL1, impaired ipRGC light perception) may lead to ocular rhythm imbalance and promote myopia.

Stone and colleagues demonstrated that core circadian clock genes expressed in the retina and choroid display distinct circadian oscillation patterns in animal models, whose expression patterns are closely correlated with changes in axial growth rate and refractive status (Stone et al., 2024b). These rhythmic expressions may participate in the regulation of axial growth through multiple mechanisms in animal models, with direct evidence in humans still lacking.

Additionally, circadian rhythms potentially play a role in the onset and progression of myopia by regulating the secretion and activity of various signaling molecules. The most notable of these are dopamine and melatonin. Dopamine exhibits higher daytime levels and exerts a protective effect by inhibiting excessive eyeball elongation. Melatonin peaks at night and is closely associated with sleep regulation and ocular metabolism (Feldkaemper and Schaeffel, 2013). Research indicates that disruption of the circadian rhythm alters the rhythmic secretion of dopamine and melatonin, which may contribute to imbalances in ocular signaling pathways that are potentially associated with myopia development in humans (Li et al., 2024).

The molecular basis of circadian rhythms consists of the classic molecular clock gene network. This network forms an independent, yet centrally regulated, rhythmic system within the eye. In preclinical models, these rhythms help maintain metabolic homeostasis in the eye and are closely linked to eyeball growth and refractive development through signaling pathways involving dopamine and melatonin, while human data supporting this association remain preliminary. This provides a molecular foundation for understanding the role of circadian rhythms in myopia pathogenesis.

#### 2.1.2 ipRGCs and melanopsin

As a specialized subset of retinal ganglion cells, intrinsic photosensitive retinal ganglion cells (ipRGCs) are defined by their ability to express melanopsin, an atypical light-sensitive pigment (Berson et al., 2002). Unlike traditional cone and rod photoreceptors, ipRGCs respond independently to light. They demonstrate peak light sensitivity toward blue wavelengths at roughly 480 nm, which in turn allows them to fulfill a core function in non-image-forming visual processes (Do and Yau, 2010) (including circadian rhythm light synchronization (Tu et al., 2005; LeGates et al., 2014) and the pupillary light reflex (Lucas et al., 2003; Valdez et al., 2009; Douglas, 2018).

Melanopsin belongs to the rhodopsin family and maintains a steady-state response under continuous illumination (Bailes and Lucas, 2013). Consequently, ipRGCs reliably transmit light signals to the SCN in the hypothalamus, forming the molecular basis for synchronizing mammalian circadian rhythms with external photoperiods. Simultaneously, ipRGCs project to the pineal gland and other hypothalamic regions, indirectly influencing the rhythmic secretion of melatonin. They also interact with the retinal dopamine system, establishing a “light-melatonin-dopamine” regulatory circuit (Li et al., 2024).

Recent findings have indicated that the involvement of intrinsically photosensitive retinal ganglion cells (ipRGCs) and melanopsin in the development and progression of myopia is becoming increasingly evident in animal models, while human evidence remains correlational. On the one hand, blue light-induced activation of ipRGCs has been found to modify the transient changes in axial length of the eye in small-scale human studies and animal models, a finding that suggests a potential regulatory impact on ocular morphology (Chakraborty et al., 2023). On the other hand, dysfunction in the phototransduction pathway associated with melanopsin may disrupt the circadian rhythmic secretion of dopamine and melatonin in animal models, which could potentially upset the developmental equilibrium of the eyeball (Amorim-de-Sousa et al., 2024). These findings suggest that ipRGCs/melanopsin not only serve as a core photoreceptive mechanism for circadian rhythms but may also link environmental light changes to myopia susceptibility in animal models; human evidence remains correlational.

#### 2.1.3 Potential effects of circadian dysregulation on eye development

The precise operation of circadian rhythms is crucial for maintaining normal eye development. Disruption in the expression of clock genes or their synchronizing mechanisms may lead to metabolic and growth dysregulation in ocular tissues (Takahashi, 2017). Research has indicated that the aberrant expression of core clock genes (including BMAL1, PER, and CRY) is associated with systemic metabolic disorders and disrupts peripheral circadian rhythms in the eye in animal models. This disruption may impair the circadian rhythmicity of retinal and choroidal activities, as observed in preclinical studies (McMahon et al., 2014; Stone et al., 2024b).

In the retina, circadian rhythm disruption interferes with the rhythmic secretion of dopamine. As a protective factor against myopia in animal models, dopamine typically peaks during the day, but circadian rhythm disorders reduce its daytime levels in preclinical studies, which may weaken its inhibitory effect on excessive elongation of the eyeball—though direct human evidence for this mechanism is limited (Feldkaemper and Schaeffel, 2013; Li et al., 2024). Simultaneously, the secretion rhythm of melatonin is also disrupted. Nighttime exposure to light or insufficient sleep has been shown to lead to a rhythmic decline in melatonin levels in humans and animals, which may potentially exacerbate the risk of myopia by disrupting the synchrony of the retinal-pineal axis (Liu et al., 2023; Dong et al., 2024).

Furthermore, ipRGCs and their melanopsin pathway, serving as the bridge between light input and the central clock, also exhibit dysfunction under circadian misalignment. For instance, excessive nocturnal blue light exposure can cause ipRGC-mediated light signals to become desynchronized from SCN rhythms in animal models, creating a “phase mismatch” between peripheral and central circadian clocks. This may further exacerbate myopia-related abnormalities in ocular axis regulation (Chakraborty et al., 2023; Amorim-de-Sousa et al., 2024). The choroid and sclera, as crucial peripheral tissues for ocular growth, exhibit circadian fluctuations in both thickness and matrix metabolism. Disruption of the biological clock can lead to an imbalance in the structural remodeling processes of these tissues in animal models, which may thereby promote axial elongation of the eye (Nickla, 2006).

Circadian rhythm disruption may disrupt the dynamic equilibrium of eye development through multiple pathways in animal models, including the disruption of dopamine and melatonin rhythms, impaired light input to ipRGCs, and abnormal expression of peripheral circadian clock genes in the eye. This understanding not only integrates the roles of the molecular clock, light signaling pathways, and neurotransmitter rhythms but also provides a molecular basis for understanding the role of environmental light and altered sleep rhythms in the development of myopia.

### 2.2 The role of circadian rhythm dysregulation in myopia pathogenesis

#### 2.2.1 Effects of altered light rhythms on myopia

Light represents the primary exogenous factor regulating circadian rhythms, and its influence on ocular development has been extensively documented. Via ipRGCs, light stimuli are transmitted to the hypothalamic SCN, which serves to synchronize the molecular clocks present in peripheral tissues in animals, thereby helping preserve the rhythmic developmental growth of the eye (Herzog, 2007). similar regulatory mechanisms are hypothesized in humans but remain to be fully validated.

Firstly, alterations in light intensity and duration have been demonstrated to be closely linked to the risk of developing myopia. A body of epidemiological evidence indicates that an increase in the amount of time spent by children outdoors during the day has a significant impact on the incidence of myopia. This phenomenon is hypothesized to be associated with high-intensity natural light stimulating dopamine release in the retina in animal models, which may thereby inhibit excessive eyeball elongation—consistent with epidemiological evidence linking outdoor light exposure to reduced myopia risk (French et al., 2013; Wu et al., 2013). Conversely, excessive nighttime illumination or prolonged artificial lighting may disrupt the rhythmic regulation of the eyeball in humans, while animal studies have shown that continuous exposure to light can cause abnormal elongation of the eyeball (Jensen and Matson, 1957; Li et al., 1995). Secondly, spectral characteristics also play a role in regulating myopia. Blue light is the primary stimulating wavelength for ipRGCs and melanopsin. Exposure to blue light during nighttime hours suppresses the secretion of melatonin in humans and induces a state of desynchrony between the central circadian clock and its peripheral counterparts in animal models, which may consequently elevate the susceptibility to myopia (Menéndez-Velázquez et al., 2022).

Furthermore, the timing of light exposure is critically important. Nighttime light exposure (especially before bedtime) significantly suppresses melatonin secretion in humans and disrupts the sleep-wake cycle as well as the molecular circadian rhythms of the eye in animal models, which may in turn elevate the susceptibility to myopia (Cajochen et al., 2011). Conversely, daytime or early morning light exposure helps “calibrate” the biological clock, maintaining the normal rhythmic changes of the eyeball and sclera (Ostrin, 2019).

These findings suggest that changes in modern environmental light patterns may represent a significant external driver of the myopia epidemic.

#### 2.2.2 Effects of sleep-wake cycle alterations on myopia

The sleep-wake cycle is a key phenotype of circadian rhythms. Its disruption is not only associated with systemic diseases but is increasingly recognized as a risk factor for the onset and progression of myopia. Normal sleep helps maintain the nocturnal peak secretion of melatonin and synchronizes ocular rhythms in animals and humans. However, this equilibrium may be disrupted by sleep deprivation, delayed sleep onset, or circadian inversion (Potter et al., 2016).

Research on the association between childhood sleep and myopia has yielded conflicting results. In the early cohort studies conducted from the 1970s to the 1990s, the utilization of the Children’s Sleep Habits Questionnaire (CSHQ) yielded no statistically significant correlation between sleep duration and quality with respect to refractive status (Zhou et al., 2015). A subsequent 20-year follow-up study by Stafford-Bell corroborated the findings, confirming the absence of a substantial association between sleep-related behaviors and alterations in refractive error, axial length, or corneal curvature radius (Stafford-Bell et al., 2022). However, subsequent research by Xu et al (Xu et al., 2023). A cross-sectional analysis of 30,188 students was conducted, and it was found that irregular sleep-wake cycles were significantly associated with an increased risk of myopia. The prevalence of myopia increased in a stepwise manner with school age (25.6% in elementary school, 62.4% in middle school, and 75.7% in high school). This discrepancy may arise from three key factors. First, early studies inadequately controlled for confounding variables, particularly outdoor light exposure, which independently improves sleep and protects against myopia. Second, inconsistent assessment metrics exist: earlier work focused solely on sleep duration, whereas recent investigations highlight the importance of sleep rhythm stability (e.g., bedtime variability). Third, population heterogeneity contributes: Asian children experience heavier academic pressure, resulting in a significantly stronger association between irregular sleep and myopia than observed in European and American children.

Animal studies also provide supporting evidence. Animal models suggest that sleep deprivation may induce ocular damage: through experiments with chronically sleep-deprived (CSD) mice, Huang’s team discovered significant remodeling of the retinal circadian rhythm transcriptome, abnormal temporal patterns in metabolic and immune-related pathways, accompanied by reactive oxygen species accumulation and rhythmic alterations in ganglion cell complex thickness (Huang et al., 2024).

Altered sleep-wake cycles have emerged as a potential environmental risk factor for myopia, supported by observational and preclinical evidence, with their mechanisms highly intertwined with circadian rhythms. Longitudinal investigations and intervention trials conducted in future research will help elucidate whether the optimization of sleep patterns can act as an innovative approach for the prevention and management of myopia.

#### 2.2.3 The causal relationship between sleep and myopia

Although an increasing number of studies have suggested associations between sleep behaviors, circadian rhythm characteristics and myopia, the causal direction underlying this relationship remains unclear. Current evidence, primarily from epidemiological studies and experimental models, suggests that the association is likely bidirectional rather than a unidirectional causal pathway.

Several population-based studies have reported that shorter sleep duration, delayed bedtime, and evening circadian preference are associated with a higher prevalence or greater severity of myopia (Saara et al., 2022; Wang et al., 2022). In addition, it has been proposed that irregular sleep patterns may indirectly contribute to refractive development by modulating the dopamine-melatonin secretion rhythm and diurnal variations in ocular axial length (Liu et al., 2025). However, most of these studies employed cross-sectional or observational designs, which cannot confirm whether sleep disturbances directly drive the development of myopia but only suggest a potential association.

On the other hand, myopia-related alterations in retinal structure and neural signaling may theoretically exert reverse effects on the circadian rhythm system. Animal studies have demonstrated that changes in retinal neurotransmitters and light signal processing can modulate the phase of circadian rhythms (Ruan et al., 2008). In humans, several studies have observed shifted sleep rhythms or altered melatonin rhythms in individuals with myopia (Chakraborty et al., 2021; Chakraborty et al., 2024).

Similar phenomena have also been reported in other retinal diseases. For instance, altered circadian preference has been documented in patients with age-related macular degeneration, suggesting that retinal dysfunction itself may impact the central circadian system (Garces et al., 2025). These findings suggest that the retina-circadian axis may play a role in multiple ocular diseases, not limited to myopia alone.

Taking all available evidence together, a more reasonable interpretation is that there is a bidirectional interaction between sleep–circadian rhythms and myopia. On the one hand, light exposure timing, sleep patterns, and melatonin rhythms may participate in the regulation of axial length by affecting the retina–choroid–sclera signaling pathway. On the other hand, alterations in ocular growth and retinal neural activity may also exert feedback effects on the circadian regulatory network. This bidirectional model can account for inconsistent findings across studies and highlights the need for future longitudinal studies and mechanistic investigations to clarify the relative contribution of each pathway.

### 2.3 Species differences between human and animal models in myopia research

When interpreting the potential role of circadian rhythms in the onset and progression of myopia, sufficient consideration should be given to differences in ocular structure and physiological regulation across species. Current mechanistic evidence is mainly derived from experimental models including chickens, rodents, and non-human primates, whereas most human studies are observational or correlational in design. Therefore, caution should be exercised when extrapolating findings from animal models to humans (Troilo et al., 2019).

There are significant differences in the scleral tissue structure and biomechanical properties among different species, which may influence the visual signal-driven axial elongation process. The avian sclera consists of both a fibrous layer and a cartilaginous layer, whereas the mammalian sclera is mainly composed of collagen fibers and fibroblasts, with distinct rates of extracellular matrix turnover and mechanical response characteristics (McBrien and Gentle, 2003; Rada et al., 2006). These structural differences may lead to variations in the speed and magnitude of scleral remodeling in experimental myopia models, thereby amplifying or attenuating the effects of circadian rhythm signals on ocular axial growth.

The choroid exhibits distinct rhythmic dynamic changes across species. In the chick model, choroidal thickness can undergo substantial short-term variations, which are regarded as a mechanism for rapid defocus compensation (Nickla and Wallman, 2010). In contrast, although diurnal rhythmic fluctuations also exist in humans and primates, they present a smaller amplitude and are more likely to contribute to long-term axial regulation rather than immediate compensatory responses (Chakraborty et al., 2011; Ulaganathan et al., 2019). Therefore, the physiological significance of the marked choroidal rhythmic responses observed in animal models may differ in humans.

Intrinsically photosensitive retinal ganglion cells (ipRGCs) are key cell types mediating non-image-forming visual responses. Their numerical proportion, subtype composition, and central projections vary across species (Hattar et al., 2002; Schmidt et al., 2011). Such differences may influence the strength of light effects on melatonin secretion, sleep rhythms, and downstream regulatory pathways of ocular growth, thereby contributing to inconsistencies between experimental animal findings and human epidemiological studies (Lazzerini Ospri et al., 2017).

These observations suggest that the circadian rhythm-myopia regulatory mechanisms revealed by current animal studies should be regarded as biologically plausible potential pathways, rather than causal relationships conclusively verified in humans. Further longitudinal and mechanistic population studies are still needed to clarify the actual magnitude of these rhythmic pathways across species.

## 3 The relationship between circadian rhythms of ocular tissue and myopia

**Figure 2:**
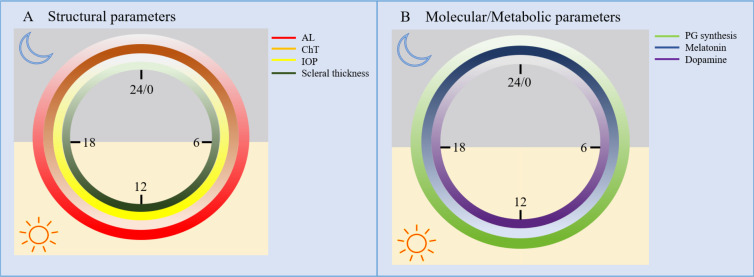
Circadian rhythms of ocular refractive biological parameters and key neurotransmitters. **(A)** Structural parameters: AL (axial length, red), ChT (choroidal thickness, orange), IOP (intraocular pressure, yellow), scleral thickness (dark green); **(B)** Molecular/metabolic parameters: PG synthesis (scleral proteoglycan synthesis rate, light green), melatonin (blue), dopamine (purple). Color intensity indicates relative value (darker = higher thickness/concentration/synthesis rate).

Ocular tissues exhibit relatively independent peripheral circadian rhythms. These local clocks participate in regulating axial growth and refractive status through periodic gene expression, neurotransmitter secretion, and structural changes (McMahon et al., 2014). For instance, the retinal biological clock governs the circadian release of dopamine, while the choroid and sclera demonstrate periodic alterations in thickness and matrix metabolism. Intraocular pressure also exhibits distinct diurnal fluctuations (Stone et al., 2024b). (**Figure 2**)

**Figure 2** presents the circadian rhythmic characteristics of key ocular refractive parameters, divided into structural and molecular/metabolic parameters to clarify their temporal correlation. As shown in **Figure 2A**, axial length (AL) exhibits a diurnal rhythm of “lengthening during the day and shortening at night”, which is inversely coupled with choroidal thickness (ChT: thinning during the day and thickening at night) — this coupling forms a buffering system for short-term refractive homeostasis. Intraocular pressure (IOP) and scleral thickness also show diurnal fluctuations, with peaks during the daytime. **Figure 2B** shows that the synthesis rate of scleral proteoglycans (PG) is higher during the day, consistent with the rhythm of scleral thickness; dopamine (a myopia-protective factor) peaks during the day, while melatonin (associated with ocular metabolism) peaks at night, forming a mirror-symmetric antagonistic relationship.

Therefore, systematically investigating the mechanisms by which circadian rhythms and their disruptions in the eye influence myopia from a multi-level perspective encompassing organization, molecules, and function will not only help reveal the underlying biological basis for the onset and progression of myopia but also provide crucial theoretical foundations for designing future intervention strategies.

### 3.1 Circadian rhythm of the eye axis

Extensive preclinical and clinical research demonstrates that axial length (AL) exhibits stable circadian oscillations (lengthening during the day and shortening at night) in both animals and humans. This rhythm shows a distinct inverse relationship with choroidal thickness (ChT) rhythm (i.e., when AL is longer, ChT is thinner, and vice versa), suggesting that the eyeball and choroid may function as an integrated buffering system in short-period refractive homeostasis regulation (Nickla et al., 1998a; Stone et al., 2004; Read et al., 2008; Brown et al., 2009; Chakraborty et al., 2011; Tan et al., 2012). Beyond the “axial length-choroid” coupling, parameters such as intraocular pressure and perfusion also correlate with subtle fluctuations in axial length, suggesting that mechanical and hemodynamic effects may jointly shape short-term axial length rhythms and long-term growth trends by altering choroidal volume and scleral stress (Chakraborty et al., 2011; Tan et al., 2012; Burfield et al., 2018).

In animal models, altering visual input (e.g., form deprivation, positive/negative lens induction) may modify the phase relationship between AL and ChT rhythms. Accelerated eyeball growth tends to enhance their antiphase relationship, while growth inhibition/restoration may shift them to in-phase or phase-delayed states. This association has been repeatedly validated and is considered to be one of the key rhythm-related phenotypes in myopia development/reversal (Nickla et al., 1998a; Stone et al., 2004; Nickla and Totonelly, 2016).

The evidence above demonstrates that the diurnal rhythm of the eyeball axis is genuinely present, conserved across species, inversely coupled with choroidal thickness, and modulated by light-timing and visual signals. Its phase and amplitude characteristics serve as a “temporal biomarker” for myopia susceptibility/progression, while also providing a framework for subsequent analysis of the “rhythm-growth” pathway at the retinal-choroidal-scleral level.

### 3.2 Circadian rhythms of the retina and refractive development

Evidence has confirmed that the retina harbors inherent circadian rhythmicity in animals and humans, which implies the presence of a self-sustained endogenous biological clock mechanism. This system has been observed to respond to regulation by the SCN, as well as to generate rhythmic signals independently, in preclinical studies (Yamazaki et al., 2000). This property enables the retina to adapt to light intensity fluctuations exceeding a millionfold within a 24-hour cycle (Guido et al., 2010), thereby optimizing visual performance under varying light conditions by coordinating circadian adjustments of visual function and metabolic activity (Yamazaki et al., 1999; Storch et al., 2007; Golombek and Rosenstein, 2010).

Within the retinal circadian regulatory network, the antagonistic mechanism of the melatonin/dopamine system is particularly important in animal models of myopia (Dubocovich, 1983; Ribelayga et al., 2004). It is well established in animal models that dopamine acts as a protective agent capable of inhibiting the abnormal overgrowth of the eye’s axial length, while human evidence supporting this protective role remains correlational. Its secretion rhythm is regulated by light intensity and the retinal clock, reaching peak secretion during the day; melatonin, conversely, rises at night and exhibits mirror-symmetric patterns with dopamine. Animal and *in vitro* studies indicate that disrupting these rhythms (e.g., prolonged nighttime illumination or abnormal light spectra/intensities) may alter dopamine signaling and promote abnormal eyeball growth (Sakamoto et al., 2005; Ruan et al., 2008; Feldkaemper and Schaeffel, 2013; Stone et al., 2013).

As light-sensitive cells, ipRGCs not only drive pupillary reflexes synchronized with circadian rhythms but also regulate neurochemical homeostasis within the retina. Recent studies in both animals and humans have revealed that ipRGC activity is closely linked to recent light exposure. Research further suggests that alterations in ipRGC signaling may be associated with refractive status (though population findings are not entirely consistent, necessitating stratified analysis by age, light exposure history, and other factors) (Lan et al., 2014; Ostrin, 2018; Chakraborty et al., 2022; Liu et al., 2022).

Epidemiological and intervention studies provide macro-level support for the “light-circadian rhythm-myopia” association. Multiple large-scale cohort and randomized trials demonstrate that increased outdoor light exposure reduces myopia incidence. Recent concerns regarding nocturnal artificial light exposure/LEDs have also proposed tentative evidence suggesting that nighttime light pollution may disrupt retinal melatonin/dopamine rhythms, thereby affecting refractive development (Ashby et al., 2009; Lan et al., 2014; He et al., 2015; Chakraborty et al., 2018; Zhang et al., 2023).

The retina serves as both a hub for light-sensing and circadian clock inputs, and transmits light/time signals to downstream tissues through rhythmic neurotransmitters (dopamine/melatonin), time-of-day-dependent gene expression, and diurnal morphological changes (choroid, axial length), thereby participating in the phenotypic expression of refractive regulation and myopia development. Existing data collectively suggest that the development of myopia may be associated with abnormalities in the retinal circadian rhythm regulatory network, though a direct causal link remains unproven.

### 3.3 Circadian rhythms of the choroid and sclera

The choroid in humans and various animals exhibits distinct circadian variations, typically being thinner during the day and thicker at night. This rhythm shows an inverse relationship with eyeball length fluctuations (the choroid is thinnest when the eyeball reaches its maximum length during the day) (Nickla et al., 1998a; Nickla, 2006; Read et al., 2008; Nickla, 2013). Under refractive intervention conditions (such as form-deprivation or negative lens induction), the circadian phase and amplitude of the choroid undergo predictable alterations in animal models: Rapid growth or myopic conditions often cause phase advancement or amplitude changes, while inhibiting eyeball elongation (such as applying myopic defocus) can trigger choroidal phase responses and predict subsequent growth rate changes in preclinical studies. These dynamic changes have been repeatedly validated in avian and primate models, suggesting that the choroid serves not merely as a passive volume buffer but as a potential relay station for growth “signals” emitted by the retina and their short-term biological responses (Ashby and Schaeffel, 2010; Lan et al., 2013; Nickla and Totonelly, 2016; Ulaganathan et al., 2019).

In human clinical studies, myopic individuals often exhibit greater diurnal fluctuations in axial length and thinner mean choroidal thickness, with diurnal amplitude showing a potential significant correlation to subsequent axial length changes over the following year or even years. Cohort and intervention studies indicate that increased outdoor high-intensity light exposure or specific optical/phototherapy interventions (such as repeated low-intensity red light) may induce short-term choroidal thickness (ChT) increase in humans, which may predict long-term efficacy in myopia control (McBrien and Gentle, 2003; McGlinn et al., 2007; Moderiano et al., 2019; Ostrin et al., 2023; Chen et al., 2024). Animal studies have also revealed that nocturnal illumination, alterations in light spectrum/intensity, or light exposure at different times of day may rapidly disrupt the circadian rhythm of the choroid (including suppression of rhythmicity or phase shifts), accompanied by long-term changes in the pattern of eyeball growth. This indicates that light-rhythm input can influence the trajectory of ocular growth via the choroid in animal models, with human relevance yet to be fully confirmed (Nickla et al., 2017; Sarfare et al., 2020; Ostrin et al., 2023; Shen et al., 2024).

The sclera serves as the terminal effector tissue in myopia progression, with scleral extracellular matrix (ECM) remodeling directly determining its biomechanical properties and resistance to axial elongation. Research indicates that chick eye axis growth is influenced by the synthesis rate of scleral proteoglycans (matrix molecules) (Rada et al., 1991; Rada et al., 1992; Rada and Matthews, 1994; Rada et al., 1994; Nickla et al., 1997), suggesting that rhythmic scleral matrix synthesis may underlie periodic variations in eye axis length.

Furthermore, research indicates that the synthesis of scleral proteoglycans exhibits significant circadian fluctuations, with a significantly higher synthesis rate during the day than at night. This rhythm can be autonomously sustained for a minimum of three cycles *in vitro*, with no discernible difference observed between normal eyes and form-deprived eyes (Nickla et al., 1999). The thickness of the temporal anterior sclera in humans also exhibits rhythmic changes, reaching its maximum in the early morning (21μm thicker than at noon) (Read et al., 2016). This suggests the potential involvement of circadian mechanical stress in regulating scleral biomechanical properties.

Experimental models reveal the impact of external circadian rhythm disruption. In BMAL1 knockout mice and the light-on-night (LAN) model, reduced ECM synthesis in the sclera may leads to decreased matrix strength (Chen et al., 2023). It is noteworthy that excessive elongation of the eyeball during myopia progression may be associated with phase shifts in scleral rhythmic parameters (rather than changes in amplitude). The circadian rhythm governing proteoglycan synthesis does not exhibit any statistically meaningful differences when comparing the sclerae from normal individuals and myopic subjects. However, persistent rhythmic disturbances (such as LAN) may disrupt scleral homeostasis through cumulative effects in animal models.

### 3.4 Association between intraocular pressure rhythm and myopia

The circadian rhythm fluctuations of intraocular pressure (IOP) are closely related to the dynamic equilibrium of aqueous humor circulation (Ikegami, 2024). Clinical observations indicate that myopic individuals may exhibit greater diurnal fluctuations in intraocular pressure (e.g., significantly elevated daytime IOP in myopic glaucoma patients), but the role of these fluctuations in myopia progression remains unclear (Jeong et al., 2014). Cross-sectional studies confirm significant differences in IOP, AL, and refractive error between the myopic and emmetropic groups (Lu et al., 2022). However, longitudinal studies yielded conflicting results. Wilson et al. found no direct association between diurnal fluctuations in IOP and changes in AL (r² < 0.15) (Wilson et al., 2006). Li reported in a three-year cohort study that myopia progression in school-aged children was negatively correlated with baseline IOP (Li et al., 2019), suggesting that myopia progression in this age group is largely independent of IOP. IOP may indirectly influence the progression process through alterations in scleral biomechanical properties in animal models, though human studies have shown weak correlations.

Animal studies have elucidated the mechanism underlying IOP circadian rhythms. In the chick model, diurnal IOP fluctuations may directly induce axial length elongation through mechanical stress while also potentially influencing extracellular matrix remodeling by regulating metabolic activity in scleral fibroblasts (such as the rhythmic secretion of MMP-2) (Nickla et al., 1998b).

In high myopia and pathological myopia, the circadian rhythm of IOP often exhibits delayed nocturnal peaks or increased amplitude. This rhythmic high load may impose repetitive mechanical stress on the posterior sclera, thereby potentially promoting extracellular matrix (ECM) remodeling and accelerating the pathological process of axial elongation (Wang et al., 2021). Animal and gene knockout studies further suggest that disruption of circadian rhythm genes may alter ocular tissue development and matrix homeostasis (e.g., retinal Bmal1 knockout induces axial elongation), indicating that rhythm disruption may influence growth signaling through neurochemical pathways while also potentially amplifying the pathogenic effects of mechanical stimuli by altering IOP rhythms (Stone et al., 2019; Stone et al., 2024a).

Mechanical-biochemical coupling serves as the critical bridge linking IOP rhythms to pathological myopia. Periodic or sustained elevated nocturnal IOP activates mechanical transduction pathways (such as YAP/TAZ and downstream Smad/TGF-β signaling) in animal models, driving phenotypic alterations in scleral fibroblasts and shifting the MMP/TIMP balance toward the degradative side. This process may subsequently weakens collagen synthesis and diminishes scleral mechanical strength. Simultaneously, the imbalance in core circadian gene expression and the upregulation of pro-inflammatory/hypoxic signaling within the diseased sclera may further impair adaptive repair to stress rhythms. This creates a potential vicious cycle where mechanical rhythms compound molecular rhythms, ultimately potentially promoting the development and progression of high/pathological myopia (Hu et al., 2021; Yu and Zhou, 2022; Ku et al., 2024).

The above findings connect with the scleral remodeling mechanism described in Section 3.3. IOP rhythms do not directly drive axial length growth but instead regulate refractive development by altering scleral biomechanical properties (such as thickness and elastic modulus). This mechanistic framework explains the weak correlation between IOP and AL growth observed in human studies while providing theoretical support for intervention strategies targeting biomechanical regulation.

### 3.5 Coupling of circadian rhythm networks with myopia progression

Under physiological conditions, the circadian rhythm regulatory network of ocular tissues helps maintains the temporal coordination of eye development and visual function in animals and humans. However, when any component of the system (such as retinal dopamine rhythms, the light-dark cycle, or diurnal fluctuations in intraocular pressure) becomes disrupted or weakened, the overall coupling may be interrupted. This disruption may create potential favorable conditions for the onset and progression of myopia (Stone et al., 1989; Iuvone et al., 1991; Read et al., 2008; Feldkaemper and Schaeffel, 2013; Nickla and Totonelly, 2016).

Specifically: Rhythmic signals at the retinal level (such as circadian oscillations of dopamine and melatonin) may serve as upstream rhythmic “triggers” and synchronizers. Their disruption may alter the short-term volume/blood flow rhythms of the choroid and the circadian regulation of the scleral ECM in animal models, thereby potentially influencing both the daily and long-term growth patterns of the eyeball (Feldkaemper and Schaeffel, 2013; Lan et al., 2013; Shen et al., 2024). Concurrently, the diurnal fluctuations of IOP, along with its phase and amplitude, may also contribute to the dynamic equilibrium of the axial length through mechanical tension and scleral compliance. Should the IOP rhythm be weakened or its phase misaligned, the long-term accumulation of mechanical stress may promote scleral remodeling and axial length elongation (Brubaker, 1991; Liu et al., 2002; Loewen et al., 2010). Multiple transcriptomic and time-dependent gene expression studies further suggest that numerous pathways associated with ECM regulation, vascularization/perfusion, and circadian genes exhibit time-dependent alterations in myopia induction models, emphasizing “time” as a potentially critical experimental and interpretive variable (Moderiano et al., 2019; Stone et al., 2024b).

Therefore, we believe subsequent research should focus on two key aspects: First, treating time (the temporal phase of sampling/measurement) as a fundamental variable—any ocular biological measurement should concurrently report both the time-of-day and the subject’s light exposure history. Second, adopting cross-tissue, multi-parameter synchronous monitoring and analysis (e.g., simultaneously recording AL, ChT, IOP, and retinal/choroidal molecular circadian rhythms) to elucidate how failed rhythmic coupling translates into structural changes associated with myopia. choroid molecular circadian rhythms) to elucidate how failed rhythmic coupling translates into structural changes associated with myopia. This research strategy will provide essential chronological data support for integrating the mechanisms underlying circadian rhythm-induced myopia, while also guiding the design of future rhythm-based intervention studies (such as time-pacing phototherapy and sleep rhythm adjustment).

## 4 Integration of potential mechanisms and molecular pathways

We will first summarize how light signals (particularly those mediated by ipRGCs/rhodopsin) synchronize retinal and central clocks and influence downstream neurochemical environments (Berson et al., 2002; Panda et al., 2003; Do and Yau, 2010), then examine the expression and regulatory roles of core circadian genes in ocular tissues and their rhythmic control of ECM and cellular metabolism (Stone et al., 2013; McMahon et al., 2014). We will emphasize integrating dopamine-melatonin and other neurotransmitter/hormone pathways with the retinal-choroidal-scleral signaling cascade, ultimately proposing a testable molecular-tissue integration model to provide mechanistic foundations for subsequent rhythm-based intervention strategies (Read et al., 2008; Feldkaemper and Schaeffel, 2013; Wang et al., 2024).

### 4.1 Light signal perception and transmission

Light serves as the primary exogenous zeitgeber, regulating circadian rhythms through the non-imaging visual pathway of the retina.

Experimental studies indicate that non-imaging photoreactions are severely impaired in melanopsin-deficient mice, manifesting as circadian rhythm disruption and abnormal retinal dopamine levels (Panda et al., 2003; McMahon et al., 2014). In avian and mammalian models, increased light intensity may suppresses myopic eye elongation, whereas prolonged low-light or nocturnal light exposure may disrupts ocular circadian rhythms and promotes myopia development (Ashby and Schaeffel, 2010; Lan et al., 2013; Nickla and Totonelly, 2016). For example, studies in chicken models demonstrate that intense light significantly increases choroidal thickness and may suppresses myopia progression (Stone et al., 2013), while brief nocturnal illumination can disrupt the circadian oscillations of the eyeball in avian models (Chakraborty et al., 2018).

Regarding evidence from human cohorts, large-scale clinical research has found that extending outdoor exposure duration is effective in decreasing the likelihood of myopia development, suggesting that the light signaling pathway plays an important role in ocular refractive maturation (He et al., 2015). Further research conducted on pediatric populations indicates that pupillary responses mediated by ipRGCs are correlated with ocular refractive status, which in turn underscores the potential contribution of non-image-forming visual pathways to myopia pathogenesis (Ostrin, 2018). Recent studies have even discovered that blue light stimulation of the blind spot region can activate circadian-related physiological responses via ipRGCs in human volunteers, suggesting a potential link between ipRGC dysfunction and myopia risk that requires further validation in large-scale cohort studies (Amorim-de-Sousa et al., 2024; Li et al., 2024).

Therefore, the intensity of light signals, their spectral composition, and the phase of exposure collectively determine the activity of ipRGCs/Melanopsin in animal models. These signals synchronize molecular clock genes with neurotransmitter rhythms, ultimately regulating the rhythmic remodeling of ocular tissues (Takahashi, 2017). This segment serves as the “input terminal” of the entire circadian rhythm network, providing the essential prerequisite for subsequent investigations into the roles of molecular clock genes and neurotransmitter pathways in the development of myopia.

### 4.2 Clock genes and myopia progression

The clock genes within the retina constitute a crucial component in the mechanism of myopia development. Core clock factors BMAL1 and CLOCK form a classic transcription-translation negative feedback loop by transcriptionally activating PER and CRY, thereby helping maintaining the stability of circadian rhythms (Storch et al., 2007). Animal studies indicate that retinal-specific knockout of BMAL1 may induces photoreceptor dysfunction and structural abnormalities, accompanied by disrupted refractive development (Baba et al., 2018). At the population genetics level, clock gene polymorphisms are significantly associated with myopia risk, suggesting that molecular rhythms may be involved in eyeball development (Kiefer et al., 2013).

Clock genes not only maintain endogenous rhythms but also participate in myopia development by regulating neurotransmitters. Research findings indicate that the cyclical expression of PER1 and CRY1 genes is tightly linked to dopamine secretion within the retina in animal models, with dopamine serving as a key factor in inhibiting abnormal axial elongation of the eye (Doyle et al., 2002; Ribelayga et al., 2004). When clock gene expression are disrupted, the amplitude of diurnal dopamine fluctuations may decreases in animal models, potentially leading to weakened inhibitory signals on eyeball elongation and thereby promoting myopia progression (Feldkaemper and Schaeffel, 2013). Additionally, clock genes and melatonin synthesis-related genes exhibit circadian-phase interactions. Abnormal melatonin levels may indirectly accelerate axial elongation by disrupting choroidal and scleral signaling pathways (Hussain et al., 2023).

Transcriptomic studies have further revealed the link between clock genes and myopia. Under conditions of visual deprivation or anisometropia, the circadian expression patterns of multiple clock genes in the retinas and choroids of chicken and mouse models were altered (Stone et al., 2011; Stone et al., 2020). These circadian-specific gene expression changes are closely associated with extracellular matrix remodeling, inflammatory signaling, and blood flow regulation, providing molecular evidence for how circadian rhythm disruption may drives tissue-level myopia phenotypes (Chakraborty et al., 2018).

Therefore, circadian clock genes may exert a pivotal role in myopia onset and progression by modulating neurotransmitter and hormonal rhythmicity, while coordinating the signaling network spanning the retina, choroid, and sclera.

### 4.3 Neurotransmitter and hormone pathways

The influence of circadian rhythms on eye development relies not only on the oscillation of clock genes but also on neurotransmitter and hormone pathways that convert light signals into chemical signals regulating eyeball growth (Green and Besharse, 2004). Animal studies indicate that blocking dopamine receptors may accelerates myopia progression, while activating the corresponding pathways can partially reverse refractive changes (Chen et al., 2017; Huang et al., 2022).

In contrast to dopamine, melatonin primarily exhibits a nocturnal secretion peak. It interacts with circadian clock genes via MT1/MT2 receptors, influencing the synthesis and remodeling of the extracellular matrix (ECM) in scleral fibroblasts, thereby promoting axial elongation of the eye (Tosini et al., 2008; Wiechmann and Summers, 2008). Clinical studies have found that individuals with myopia often exhibit elevated melatonin levels or delayed secretion phases, suggesting that this alteration is associated with the myopic phenotype (Kearney et al., 2017; Chakraborty et al., 2021). Melatonin not only directly influences scleral signals but also exerts an indirect effect by inhibiting dopamine synthesis in the retina, establishing a circadian antagonistic relationship between the two in preclinical studies (Iuvone et al., 2005; Storch et al., 2007).

The interaction between dopamine and melatonin tightly couples light signals with molecular rhythms in animal models: during the day, light exposure enhances dopamine release, potentially inhibiting excessive eye elongation, while at night, melatonin signaling dominates and may promote axial growth. This dynamic equilibrium, once disrupted by abnormal ambient light or sleep rhythm disturbances, may translate into a long-term risk of myopia in susceptible individuals (He et al., 2015; Landis et al., 2021).

**Figure 3:**
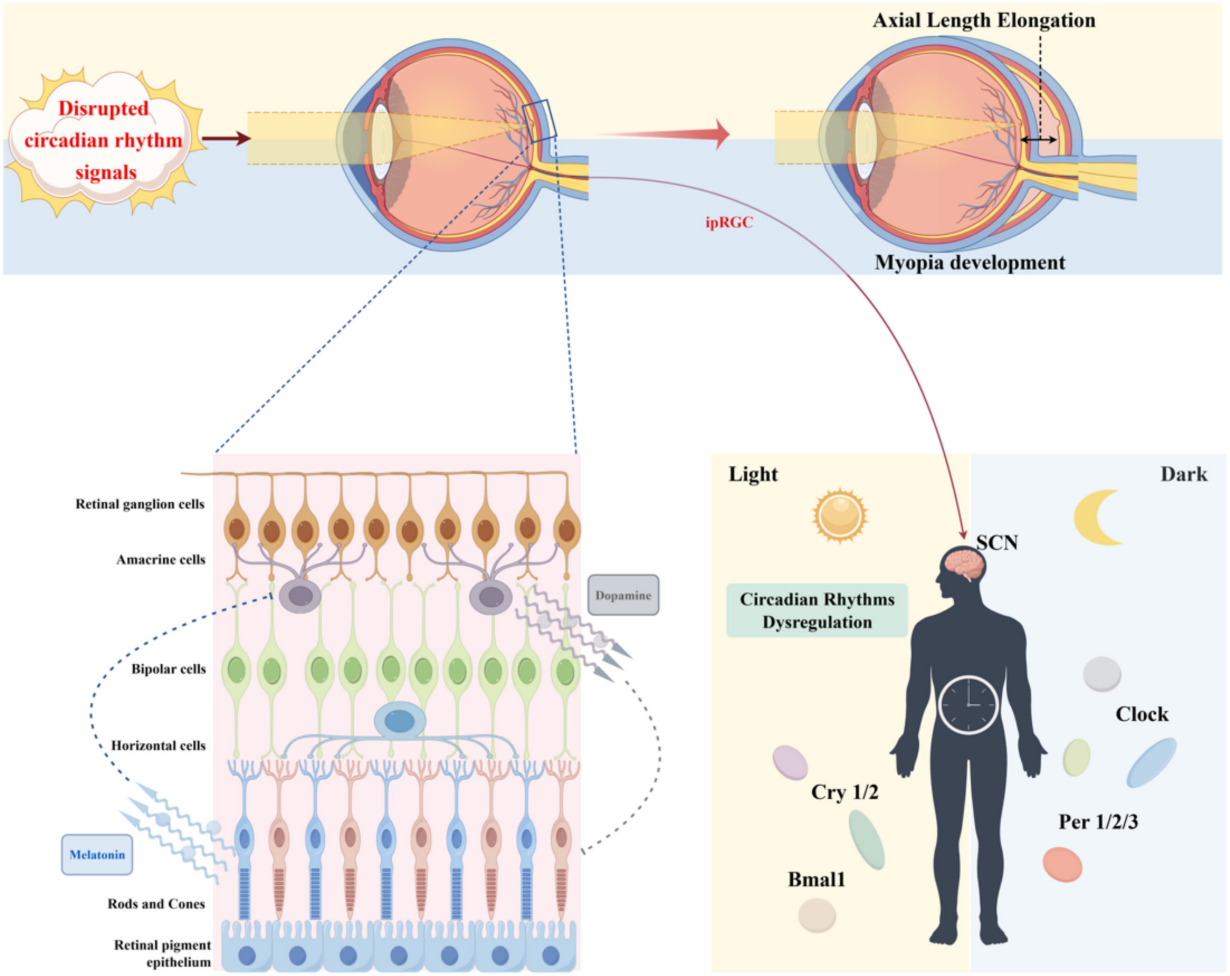
Schematic diagram of the mechanism by which circadian rhythm disruption accelerates myopia. Disrupted circadian rhythm signals (such as artificial light exposure at night) interfere with intrinsic photosensitive retinal ganglion cell signaling and circadian gene rhythms, leading to neurotransmitter imbalance and axial elongation. ipRGCs: Intrinsically photosensitive retinal ganglion cells; Clock genes: Per1/2/3, Cry1/2, BMAL1. By Figdraw.

The circadian rhythm translates the temporal sequence of external light exposure into signals for eyeball growth through a bidirectional regulatory framework involving dopamine inhibition and melatonin promotion, constituting the core neurochemical pathway governing myopia progression (**Figure 3**).

**Figure 3** illustrates the core mechanism by which circadian rhythm disruption promotes myopia progression. Under physiological conditions, the natural light-dark cycle is the basis for maintaining ocular growth homeostasis: bright daytime light activates ipRGCs, which synchronize the rhythmic expression of clock genes and enhance dopamine release to inhibit excessive axial elongation; dark nights allow for normal melatonin secretion, which regulates scleral extracellular matrix (ECM) remodeling and maintains ocular tissue rhythm.

However, modern environmental light pollution (e.g., nocturnal blue light exposure from LEDs) disrupts this equilibrium: abnormal activation of ipRGCs leads to phase mismatch between central and peripheral circadian clocks, accompanied by reduced daytime dopamine levels and delayed nighttime melatonin secretion. The weakened inhibitory effect on ocular growth and disrupted tissue homeostasis ultimately drive axial elongation and myopia development. Notably, the protective role of natural light-dark cycles in refractive development has been validated in both animal models and human epidemiological studies, while nocturnal light exposure is consistently associated with increased myopia risk.

## 5 Conclusion

The circadian rhythm, as the body’s intrinsic time-regulating system, may play an important role in the onset and progression of myopia, supported by accumulating preclinical and observational evidence. Rhythmic activities originating from ocular tissues such as the retina, choroid, and sclera, coupled with dynamic changes in signaling molecules like dopamine and melatonin, collectively influence axial length growth and refractive development in animal models, with correlational evidence observed in humans. Increasing evidence indicates that disruptions in light exposure rhythms and sleep-wake cycles may disturb the stability of ocular molecular clocks. This potential imbalance between ocular development and environmental factors accelerates myopia progression.

In-depth research on circadian rhythms not only enhances our understanding of the molecular and tissue mechanisms underlying myopia but also offers novel insights for intervention strategies. In the future, rhythm-based preventive and therapeutic measures—such as optimizing light exposure patterns, improving sleep habits, and targeted pharmacological interventions on rhythm-related signaling pathways—hold promise as potential complementary approaches for myopia control, though further clinical trials are needed to validate their efficacy. Further exploration combining clinical studies and animal experiments may help advance us from understanding the mechanisms to personalized and precision-based myopia management.

This review has certain limitations. Most regulatory mechanisms are derived from animal models, and minimally invasive techniques for the direct detection of ocular tissue circadian rhythms in humans (e.g., *in vivo* expression of retinal clock genes) remain lacking. The bidirectional causal relationship between sleep and myopia, as well as the targeted intervention of the ipRGC pathway, still require more mendelian randomization and longitudinal clinical studies. Future research may prioritize cross-species mechanism validation and rhythm-oriented myopia interventions (e.g., time-targeted phototherapy, sleep rhythm correction), with the goal of providing potential novel and precise strategies for myopia prevention and control.
